# Research note: Mobile slaughter in poultry meat production in Germany – First microbiological results

**DOI:** 10.1016/j.psj.2026.106421

**Published:** 2026-01-12

**Authors:** Melissa Bonczyk, Thomas Alter, Vanessa Szott, Katharina Charlotte Jensen, Marc Boelhauve, Antje Schönknecht

**Affiliations:** a Institute of Food Safety and Food Hygiene, School of Veterinary Medicine, Freie Universität Berlin, Königsweg 69, Berlin 14163, Germany; b Institute for Veterinary Epidemiology and Biostatistics, School of Veterinary Medicine, Freie Universität Berlin, Königsweg 67, Berlin 14163, Germany.; c Department of Agriculture, Westphalia University of Applied Sciences, Soest 59494, Germany

**Keywords:** Mobile poultry processing unit, Process hygiene, *E. coli*, Total viable count, *Campylobacter* spp.

## Abstract

Small-scale poultry farming is gaining increasing importance in Germany. At the same time, the ongoing structural transformation within the poultry processing sector has led to a growing centralization in a few large-scale slaughterhouses. As a result, many small-scale farms face challenges in having their animals processed. Mobile Poultry Processing Units (MPPUs) offer a promising solution by enabling on-site slaughter of smaller flocks, thereby significantly contributing to animal welfare by eliminating the need for long-distance transportation. However, like stationary facilities, mobile slaughter units must also comply with the stringent hygiene standards required for food processing.

In this study, we examined the development of *Campylobacter* spp., *Escherichia coli* (*E. coli*), total aerobic viable counts (TVC) and *Salmonella* spp. during the slaughter process in a MPPU in western Germany. In total, 160 neck skin samples from broiler and laying hen carcasses as well as surface swab samples, and process water (scalding tank and water bath) were examined.

A total of 76 (48 %) carcasses showed *Campylobacter* counts above the limit of detection (2.3 MPN/g; MPN = most probable number). The distribution of MPN values across processing steps indicated a gradual increase in *Campylobacter* contamination, with defeathering and evisceration identified as crucial points. Evisceration was associated with the highest intermediate contamination levels, as 26 % of samples (10/39) reaching 23 MPN/g and 18 % of samples (7/40) showing elevated counts of 230 MPN/g. A modest decrease in *Campylobacter* levels regarding intermediate and elevated counts was observed following the water bath. After scalding, *E. coli* were not detected, however, after the water bath median counts of 3.67 log CFU/ g were determined. TVC remained relatively stable, ranging between 4.79 log CFU/g (after scalding) and 4.93 log CFU/g after the water bath. *Salmonella* spp. were not detected.

This study provides insights into a mobile poultry processing unit in Germany, highlighting critical hygiene points comparable to those in conventional slaughterhouses. The findings contribute to a better understanding of microbial dynamics in MPPUs and serve as foundation for further investigations.

## Introduction

Over the past few years, the structure of poultry slaughtering has significantly changed. Smaller, regional abattoirs are progressively being replaced by larger processing facilities, resulting in a concentration of poultry processing in a few central locations across Germany (Gothe, 2019). This shift has led to longer transport routes for livestock, which are associated with increased pre-slaughter stress and a higher risk of transport injuries (Hultgren, 2019).

In contrast, alternative husbandry systems such as mobile housing systems are gaining recognition as important elements of poultry production systems. Small poultry farms, in particular, face considerable challenges in processing their products. Processing in large abattoirs is often not feasible, as they require a minimum number of animals that small-scale producers cannot provide. Conversely, on-farm slaughter conducted by the producers themselves is generally impractical due to stringent hygiene standards and a lack of specialized personnel and expertise (Gothe, 2019). This presents a specific challenge: processing poultry from small-scale farms in a manner that ensures animal welfare while maintaining economic sustainability. One potential solution is the implementation of mobile poultry processing units (MPPU). A MPPU, either as a standalone trailer or an integrated module on a truck, allows processing directly at the farm where poultry is kept and raised. The facility is divided into an unclean area for stunning, bleeding, scalding, and defeathering, and a clean area for evisceration and carcass washing. A MPPU can reduce pre-slaughter stressors, thereby improving animal welfare and meat quality by eliminating transport-related injuries and mortality risks for poultry (Mancinelli et al., 2018). Moreover, since these units generally process only one flock per day, the potential for cross contamination between batches is minimized, further improving food safety standards (Hultgren, 2019; Mancinelli et al., 2018). Under these conditions, MPPUs represent a relevant processing option for small-scale poultry production in Germany, enabling slaughtering for holdings that would otherwise lack access to suitable processing facilities (Gothe, 2019).

Regarding food safety of MPPUs, only limited data is available, indicating the need for further research to evaluate the microbial status of the products. Therefore, the purpose of this study was to examine neck skin samples (n = 160) at four significant processing steps (scalding, defeathering, evisceration, water bath) along the slaughter line in a MPPU. TVC and *E. coli* indicate the hygiene status in the slaughtering process, while *Campylobacter* spp. and *Salmonella* spp. were selected since poultry represents a common reservoir for these zoonotic bacteria (Stearns et al., 2024).

## Materials and methods

### Sampling

Two broiler flocks (2.5 months old) and two laying hen flocks (1.5 years old) were sampled during processing in a single MPPU on four non-consecutive days in Germany between May and September 2024. The chickens (170-300 per flock) were kept in either mobile stables or free-range on-farm systems and underwent feed withdrawal for 8–12 h prior slaughter. The number of chickens processed per slaughter batch ranged from 90 to 200. As only one flock was processed per day, slaughter was conducted in batch mode, with all carcasses being processed consecutively without interruptions or intermediate disinfection.

The MPPU examined in this study was divided into an unclean and a clean processing area. The unclean area comprised the stunning and bleeding stations, a scalding tank divided into two chambers by a parallel-bar separator, and a mechanical defeathering machine. Poultry were individually stunned electrically, followed by neck cutting and exsanguination on a bleeding carousel. The scalding tank was manually filled at the beginning of slaughter and subsequent refilling was possible as required during operation; however, sampling was conducted after the initial filling. After scalding, carcasses were mechanically defeathered in a rotary drum defeatherer equipped with rubber plucking fingers and subsequently transferred through a wall pass-through into the clean area, where they were re-suspended by the feet on processing shackles for evisceration. The intestinal tract was removed using an evisceration fork, and the carcass cavities were cleaned with a vacuum device, followed by removal of the neck, crop, and feet. Finally, carcasses were placed in a water bath for initial washing and chilling, which was equipped with a continuous fresh water inflow and an overflow to maintain a constant water level during processing.

Neck skin samples were collected from the carcasses at four different slaughter processing steps: after scalding, defeathering, evisceration and a pre-chilling water bath, which was also used for washing the carcasses. At each processing step, 10 consecutive neck skin samples were collected, starting with the first station at the beginning of slaughter. Carcasses were only sampled once, resulting in a total of 160 examined carcasses. For sample collection, one person held the carcass while a second person removed 10 g of neck skin using a sterile scalpel and forceps. Fresh aseptic instruments were used for each sample to prevent cross-contamination and collected neck skins were transferred into sterile plastic bags (Nasco Sampling, Pleasant Prairie, USA). Furthermore, six surface swab samples (COPAN, Murrieta, USA) and process water were taken immediately prior and after slaughter. Sterile cotton swabs, moistened with sterile phosphate buffered saline (Carl Roth, Karlsruhe, Germany) immediately before application, were taken at the following points: plucking fingers, pass-throughs from defeathering to evisceration, neck clippers, evisceration forks, working surfaces at the evisceration area, and vacuum suckers. In total, 50 ml process water was taken from the scalding tank (average temperature immediately before slaughter: 57,8°C) and the pre-chilling water tank (average temperature: 19,3°C) and transferred into sterile Falcon tubes (Greiner Bio-One, Frickenhausen, Germany). All samples were transported in a cooling box to the laboratory, refrigerated overnight before being analyzed the following day.

### Microbiological analysis

The limit of detection (LOD) was set at 2.3 log CFU/ g for TVC and *E. coli.* Regarding *Campylobacter* spp. the limit of detection was set at 2.3 MPN/ g.

***Neck skin samples****.* Semiquantitative detection of *Campylobacter* spp. was carried out based on Peh et al. (2024). For analysis, 5 g of each neck skin sample were diluted 1:10 with *Campylobacter* enrichment broth, containing Preston Broth (Oxoid, Hampshire, United-Kingdom) supplemented with 5 % horse blood (Thermo Fisher, Waltham, USA), *Campylobacter* Preston supplement (Carl Roth), and *Campylobacter*-growth-supplement (Oxoid). After homogenization for 120 sec, serial 10-fold dilutions were incubated at 37°C for 24 h under microaerobic conditions. Subsequently, 10 μl of the enriched dilution for the semiquantitative detection of *Campylobacter* spp. were streaked on modified Charcoal-Cefoperazone-Deoxycholate (mCCDA)-Agar (Oxoid) and incubated at 37°C for another 48 h under microaerobic conditions.

The remaining 5 g of the neck skin sample were diluted 1:10 with buffered peptone water (BPW) (Merck, Darmstadt, Germany) and homogenized. After preparing a 10-fold dilution series, 100 µl of the first dilution were added to 900 µl of BPW for qualitative detection of *Salmonella* spp., mixed, and incubated at 37°C for 24 h. Then, 100 µl of the enriched dilution for *Salmonella* spp. detection were dropped onto modified semi-solid Rappaport-Vassiliadis (MSRV)-Agar (Oxoid), and incubated for 48 h at 37°C. In case of an opaque zone, indicating swarming capacity, colony material was transferred to Xylose-Lysin-Desoxycholat (XLD)- (Merck) and Brilliant-Green-Phenol-Red-Lactose-Sucrose (BPLS)-Agar (Merck), and incubated at 37°C for 24 h. Suspicious colonies were further analyzed via Matrix-Assisted Laser Desorption Ionization Time-of-Flight Mass Spectrometry (MALDI-ToF/ToF ultrafleXtreme, Bruker).

For the quantitative detection of TVC and *E. coli*, 50 μl of each dilution step from the BPW were dropped onto agar plates: Plate-Count (PC)-Agar (VWR International, Leuven, Belgium) for TVC, incubated at 30°C for 48 h, and Tryptone-Bile-X-Glucuronide (TBX-)- Agar (Merck) for *E. coli*, incubated at 37°C for 24 h.

***Surface swab samples.*** Swab samples were only examined for *Campylobacter* spp*.* Swabs were transferred to 15 ml Falcon tubes (Sarstedt, Sarstedt, Germany) containing 5 ml of *Campylobacter* enrichment broth. The tubes were mixed (3 × 3 min) and serial 10-fold dilutions were prepared for *Campylobacter* detection. Samples were then processed similarly to the neck skin samples.

***Process water.*** Process water samples were only examined for *Campylobacter* spp*.* Falcon tubes containing 50 ml of the process water were centrifuged at 23°C at 7197 x g. After 10 min, the supernatant was discarded and the remaining residuum of 5 ml was resuspended with 20 ml of the corresponding liquid medium and 10-fold dilutions were prepared. Samples were then processed similarly to the neck skin samples.

***Verification of* Campylobacter *spp.*** To confirm the presence of *Campylobacter jejuni* and/ or *C. coli*, DNA was isolated from any dilution showing bacterial growth on the semiquantitative mCCDA plates, using the Chelex method as previously described by Karadas et al. (2013). Primers were designed according to Wang et al. (2002). In brief, the total volume of 25 µl PCR reaction mixture included 2 µl of DNA template, 2.5 µl of 10x PCR buffer (Qiagen, Hilden, Germany), 9.5 mM MgCl_2_, 0.6 Mm of each dNTP (Thermo Fisher Scientific, St. Leon-Rot, Germany), 0.5 U of Taq DNA polymerase (Qiagen), and a primer mix containing 12.5 pmol of *C. jejuni* primers, 25 pmol of *C. coli* primers, and 10 pmol 23S primers. After an initial denaturation step (95°C for 4 min), the PCR involved 30 cycles of denaturation (94°C for 45 s), primer annealing (60°C for 45 s), chain extension (72°C for 45 s), and a final elongation step (72°C for 5 min). PCR products were visualized after gel electrophoresis in a 3 % agarose gel stained with GR Green (Excellgen, Rockville, USA) under UV light. The highest evaluable dilution on mCCDA plates with PCR-confirmed *Campylobacter* growth was then used to calculate the MPN (most probable number) value, using an MPN table based on ISO/TS 10272-3:2010/Cor.1:2011(E).

### Statistical analysis

Data was collected in an Excel sheet and imported into IBM SPSS (version 29.0.0.0) for statistical analyses. TVC and *E. coli* counts were log transformed. Despite this transformation, normal distribution was not achieved (Shapiro-Wilk-test: p<0.01). Therefore, differences among the four stations were tested using non-parametric Kruskal-Wallis-tests. For posthoc-tests (Dunn-Bonferroni tests), Bonferroni correction was applied as p-value adjustment and the level of significance left unchanged (*p*<0.05). For the semiquantitative count of *Campylobacter*, five stages are defined (< 2.30, 2.30, 23.0, 230, 2,400) that correspond to a log transformation. For better readability, stages were defined as “below limit” (< 2.30), “low level contamination” (2.3), “intermediate contamination” (23), “elevated contamination” (230) and “substantial contamination” (2400). Differences among stages were assessed using Fisher´s exact test as 40 % of the cells had an expected frequency lower than 5. Figures were created using Graphpad-Prism Version 5.04 (Graphpad-Software., La Jolla, USA).

## Results and discussion

### Quantitative detection of TVC and *E. coli*

**Fig. 1:**
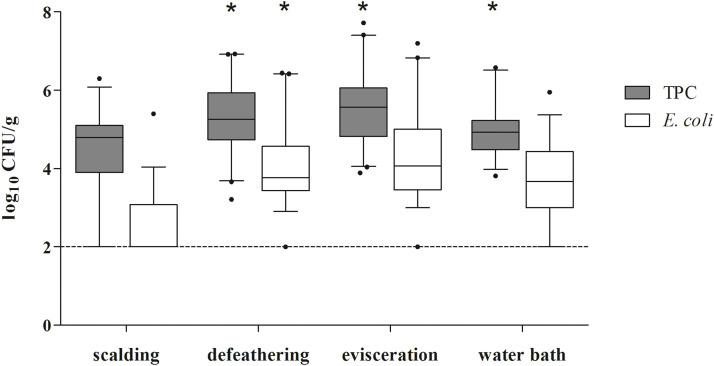
Bacterial counts for TVC and *E. coli* on neck skin samples after processing steps: scalding, defeathering, evisceration, and water bath; n = 157. Dashed horizontal line indicates the LOD. Processing steps marked with * showed a significant difference to the previous sampling point with p < 0.05.

**Fig. 2:**
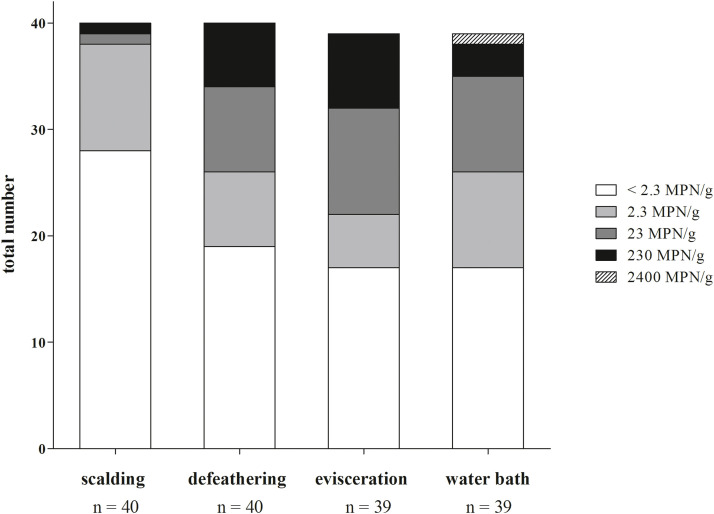
Distribution of MPN loads of *Campylobacter* spp. on neck skin samples after four different steps of poultry processing: scalding, defeathering, evisceration, pre-chilling water bath, n = 158.

In order to record the microbiological status during mobile poultry processing, chicken neck skin samples were collected after four central steps during processing (scalding, defeathering, evisceration, water bath). Due to laboratory circumstances, TVC and *E. coli* counts were determined in 157 neck skin samples (Fig. 1), while *Campylobacter* spp. counts were obtained in 158 neck skin samples (Fig. 2). *E. coli* were detected in 78 % (123/157) of the samples. During the slaughtering process examined in this study, an overall initial increase in microbial counts on neck skin samples was observed after defeathering and evisceration, followed by a decrease after the water bath.

Relative to post-scalding levels (TVC: 4.79 log CFU/ g; *E. coli*: < LOD), median counts increased significantly following defeathering, reaching 5.26 log CFU/ g for TVC and 3.77 log CFU/ g for *E. coli* (p = 0.025 / < 0.001). Regarding TVC, evisceration resulted in the maximum median value of 5.57 log CFU/ g while *E. coli* showed a nonsignificant increase of 4.01 log CFU/ g. Following the water bath, a statistically significant reduction in TVC was observed, with a median value of 4.93 log CFU/ g (p < 0.05). *E. coli* counts also declined to a final median concentration of 3.67 log CFU/ g. Another study, investigating 96 carcasses at a MPPU in the United States (Stearns et al., 2024) showed similar trends using the whole carcass rinse method. Although the sampling methodology differs from the present study, their findings are comparable with regard to the development of the microbiological status during mobile processing: microbial loads tend to increase during processing and decrease following chilling. Their reported values after immersion chilling were 4.05 log CFU/ml for aerobic plate counts and 3.46 log CFU/ml for *E. coli*, which were slightly lower than those observed in this investigation. However, our sampling concluded after the water bath, as the final chilling step takes place outside the MPPU in non-standardized private facilities that were not accessible for sampling. Therefore, microbial counts following proper chilling were not assessed. But it has been determined, that *E. coli* loads generally continue to decrease even during washing and especially after conventional chilling (Barco et al., 2014). A further decline in the contamination levels on the carcasses is therefore assumed after the final chilling step, following the processing at the MPPU examined in this investigation.

### Detection of *Campylobacter* spp.

All swab and process water samples collected prior to slaughter were tested negative for *Campylobacter* spp., indicating the absence of potential residual cross-contamination risk from previous slaughtering processes.

Post-processing, *Campylobacter* spp. were detected in 50 % (12/24) of the surface swabs. The highest proportion of positive samples was detected on the evisceration fork and on the working surface in the evisceration area, each representing 25 % (3/12). Similarly, 100 % (4/4) of the process water samples from the water bath tested positive for *Campylobacter* spp., thereby indicating a potential risk of cross-contamination (Beterams et al., 2024).

Of the 158 neck skin samples analyzed, 76 (48 %) exceeded the limit of detection (2.3 MPN/ g) for *Campylobacter* spp. Throughout the various processing steps, the prevalence of *Campylobacter*-positive carcasses increased from 28 % (11/40) after scalding to 50 % (20/40) after defeathering, reaching the highest value of 56 % (22/39) following both evisceration and water bath. This trend reflects a progressive increase in *Campylobacter* contamination throughout the slaughter process. The distribution of MPN values across processing stages (Fig. 2) further supports this observation, indicating a shift towards higher bacterial loads. Notably, the number of samples within the intermediate contamination category (23 MPN/g) increased markedly following defeathering. Only a single sample was classified in this category after scalding, whereas 20 % (8/40) of samples fell into this category after defeathering, peaking at 26 % (10/39) after evisceration. A similar trend was observed for samples with elevated *Campylobacter* levels of 230 MPN/g: only one positive sample (3 %, 1/40) was detected after scalding, increasing to 18 % (7/40) following evisceration. These results underscore defeathering and evisceration as crucial points within the slaughter process, as both steps are associated with increased *Campylobacter* contamination. In view of the limited data available on *Campylobacter* spp. in MPPUs, findings from conventional poultry slaughterhouses were considered for reference. Nevertheless, due to differing processing conditions and scale, these systems are not directly comparable, although similar process steps appear to represent hygienically crucial points. Data from conventional slaughterhouses in Germany (Beterams et al., 2024) identified defeathering and evisceration as key stages associated with an elevated risk of *Campylobacter* cross-contamination. This supports the relevance of these steps also within the MPPU. During defeathering, leakage of fecal material in combination with the mechanical forces of the plucking process has been reported to facilitate the spread of bacterial contamination, as carcasses may come into contact with feces from equipment or other carcasses. Furthermore, manual handling during evisceration may exacerbate the risk of contamination. As evisceration in the MPPU is performed manually and the extent of intermediate cleaning of tools and work gloves is not fully documented, this step may represent a potential critical point for cross-contamination within the current processing system. A considerable reduction in *Campylobacter* counts was observed between evisceration and the water bath. The proportion of samples with intermediate contamination levels of 23 MPN/ g decreased to 23 % (9/39), while those with elevated contamination levels (230 MPN/ g) declined to 8 % (3/39), highlighting the significance of the pre-chilling or rather washing step in reducing *Campylobacter* loads on carcasses. According to European legal standards defined in Regulation (EC) No. 2073/2005, process hygiene criteria are established for *Campylobacter* spp. on broiler carcasses in conventional poultry slaughterhouses. The regulation allows up to 20 % of carcasses to exceed the limit value of 1,000 CFU/ g of *Campylobacter* spp. on the carcass surface after chilling, which corresponds to 2400 MPN/ g in the present study. In our investigation, only one neck skin sample (3 %, 1/39) exceeded this threshold, indicating a generally favorable hygienic status of the chicken carcasses processed in the examined MPPU. It should be noted that these values were obtained after the pre-chilling/ washing water bath rather than after the final chilling step, and that samples originated from both laying hens and broilers, while the regulation applies exclusively to broilers.

*Salmonella* spp. were not detected in any of the samples.

In conclusion, this investigation revealed that the hygienic critical points observed in chicken processing within the MPPU are similar to those found in conventional slaughterhouses. The microbial counts after the pre-chilling/ washing water bath already appear comparable to those of conventional processing; however, no samples were collected after final chilling, potentially underestimating the actual hygienic outcome. The data obtained provide a valuable foundation for future research needed to further evaluate MPPUs as promising alternatives for poultry processing in small-scale farming in Germany, as they offer a first insight into the hygienic status of chicken and broiler carcasses processed in an MPPU and demonstrate how the main processing steps influence microbial safety.

## Funding

The project was supported by funds of the Federal Ministry of Agriculture, Food and regional Identity (BMLEH) based on a decision of the Parliament of the Federal Republic of Germany via the Federal Office for Agriculture and Food (BLE) under the innovation support programme (Project No. 281C406E21).

## Declaration of AI and AI-assisted technologies in the writing process

Statement: During the preparation of this manuscript, the authors used DeepL and ChatGPT to improve readability and language. After using these tools, the authors reviewed and edited the content as needed and take full responsibility for the accuracy and integrity of the publication.

## CRediT authorship contribution statement

**Melissa Bonczyk:** Writing – original draft, Methodology, Investigation, Formal analysis, Data curation, Conceptualization. **Thomas Alter:** Writing – review & editing, Validation, Supervision, Methodology, Funding acquisition, Conceptualization. **Vanessa Szott:** Writing – review & editing. **Katharina Charlotte Jensen:** Writing – review & editing, Formal analysis. **Marc Boelhauve:** Writing – review & editing, Funding acquisition. **Antje Schönknecht:** Writing – review & editing, Supervision, Methodology, Conceptualization.

## Disclosures

The authors declare the following financial interests/personal relationships which may be considered as potential competing interests:

Melissa Bonczyk reports financial support was provided by Federal Ministry of Agriculture, Food and regional Identity. If there are other authors, they declare that they have no known competing financial interests or personal relationships that could have appeared to influence the work reported in this paper.
